# Dr. Rogers, on the Use of Laurel Water

**Published:** 1801-06

**Authors:** J. Rogers

**Affiliations:** Great Russel Street, Bloomsbury


Gentlemen,

IN this enlightened age of ingenious inquiry and bold experiments, when medical men introduce into practice drugs, deemed by our predeceffors not only pernicious but fatally deftructive, fuch as arfenic, cicuta, belladona, digitalis, and many others, which in modified dofes, and judicious adminiftration, have by their ufe lately effected, in various cafes, extraordinary cures, it may not perhaps be thought prefumption in me to offer to the faculty another medicine, of fuch high rank in the catalogue of poifons, that the firft time I heard it propofed in confultation, I paufed, dreading its confequence.

The cafe was that of a lady, about fifty-five years old, who from the age of forty-feven had ceafed to menftruate, but from that time had been fubject to periodic difcharges of blood from the hæmorrhoidal veffels; and if at any time thofe hæmorrhages, from any caufe, were fuppreffed, violent head-ach, fpafmodic affections of the inteftines, alarming palpitation of the heart, intermittent pulfe, with rigors and cold fweats, were the confequence; thofe fymptoms ufually yielded to the application of leeches to the hæmorrhoidal veins, gentle purges, and the ufe of faline draughts. On a feverer attack than ufual, I conceived it neceffary to propofe a confultation, and a gentleman eminent in his profeffion was called in, who, after having heard the particulars of the cafe related, and examined the patient, propofed laurel water, the medicine alluded to above, of whofe innocence, in fmall dofes, he gave me the ftrongeft affurances, at the fame time inftancing its powerful effects in obftructions of the vifcera, efpecially in liver cafes. To his explanations I fubmitted

ſubmitted my faith, and agreed to its being given, the preſent caſe being an eligible preſentation for its trial; accordingly, ten drops were ordered to be taken, in a little mint water, three times a day; but the two firſt doſes producing ſo copious a perſpiration that I hardly ever before had ſeen, its further uſe was for the preſent ſuſpended.

From the time the ſweat began to break out, the patient was greatly relieved; in a ſhort time all the ſymptoms diminiſhed, and, in a few days, ſhe was reſtored to her accuſtomed ſtate of health.

From that time I loſt ſight of the lady, but believe from repeating the laurel water in very reduced doſes, with the help of bitter extracts, ſhe enjoys better health than has been her condition for ſome years paſt.

I have ſince heard that this remedy has found its way into ſome of the foreign Pharmacopœias, but do not know preciſely into which.

I was not induced to write theſe obſervations from any warranted confidence in the efficacy of the above-mentioned extraordinary active medicine, having only ſeen it given in one ſolitary inſtance; but as a hint to ſuch gentlemen who are in the habit of making experiments with heroic medicines for the benefit of mankind. I am, &c.

*Great Ruſsel Street, Bloomsbury,*

*May* 21, 1801.

J. ROGERS.

*P. S.* Since I wrote the above on laurel water, I have accidentally laid my hand on D. Jacob Reinbold Spielman's Pharmacopæia Generalis, publiſhed at Straſburgh, in 1783, in which, after claſſing and characterizing the lauroceraſus, he ſpeaks of its diſtilled water as follows: "Aquam inde diſtillatam tam brutis, quam hominibus venenatam eſſe, teſtantur obſervatores; eandem ad guttas ſexaginta aliquoties de die datam potentiſſimum viſcidi tenacis reſolvendi remedium eſſe."

---

### *To the Editors of the Medical and Phyſical Journal.*

Gentlemen,

